# Evaluation of anticonvulsant and antinociceptive properties of new N-Mannich bases derived from pyrrolidine-2,5-dione and 3-methylpyrrolidine-2,5-dione

**DOI:** 10.1007/s00210-015-1194-2

**Published:** 2015-12-09

**Authors:** Anna Rapacz, Sabina Rybka, Jolanta Obniska, Kinga Sałat, Beata Powroźnik, Elżbieta Pękala, Barbara Filipek

**Affiliations:** Department of Pharmacodynamics, Faculty of Pharmacy, Jagiellonian University Medical College, 9 Medyczna Street, 30-688 Cracow, Poland; Department of Medicinal Chemistry, Faculty of Pharmacy, Jagiellonian University Medical College, 9 Medyczna Street, 30-688 Cracow, Poland; Department of Technology and Biotechnology of Drugs, Faculty of Pharmacy, Jagiellonian University Medical College, 9 Medyczna Street, 30-688 Cracow, Poland

**Keywords:** Anticonvulsant, Antinociceptive, Mutagenicity and antimutagenicity studies, Ion channels binding, Pyrrolidine-2,5-dione, Lacosamide

## Abstract

The aim of the present experiments was to examine anticonvulsant activity of new pyrrolidine-2,5-dione and 3-methylpyrrolidine-2,5-dione derivatives in animal models of epilepsy. In addition, the possible collateral antinociceptive activity was assessed. Anticonvulsant activity was investigated in the electroconvulsive threshold (MEST) test and the pilocarpine-induced seizure models in mice. Antinociceptive activity was examined in the hot plate and the formalin tests in mice. Considering the drug safety evaluation, the *Vibrio harveyi* test was used to estimate anti/mutagenic activity. To determine the plausible mechanism of anticonvulsant action, for two chosen compounds (**12** and **23**), in vitro binding assays were carried out. All of the tested compounds revealed significant anticonvulsant activity in the MEST test. Compounds **12** and **23** displayed anticonvulsant effect also in pilocarpine-induced seizures. Four of the tested compounds (**12**, **13**, **15**, and **24**) revealed analgesic activity in the hot plate test as well as in the first phase of the formalin test, and all of them were active in the second phase of the formalin test. The possible mechanism of action of compounds **12** and **23** is the influence on the neuronal voltage-sensitive sodium and L-type calcium channels. The obtained results indicate that in the group of pyrrolidine-2,5-diones, new anticonvulsants with collateral analgesic properties can be found.

## Introduction

Epilepsy is a common neurological disorder throughout the world which is characterized by recurrent unprovoked seizures. On the one hand, preclinical research has allowed the discovery of numerous efficacious drugs. On the other hand, introduction of new drugs into clinical practice has not essentially altered the fact that seizures in 30 % of epileptics are still improperly controlled. Therefore, there is an urgent necessity for new drugs effective against drug-resistant seizures, as well as treatments that prevent or impair the process of epileptogenesis. Moreover, new therapies should particularly include drugs with improved efficacy and tolerability, since several of the currently available antiepileptic drugs (AEDs) caused severe side effects (Bialer et al. 2009; Löscher and Schmidt 2011; Simonato et al. 2014).

The mechanisms of action of AEDs are complex and include modifying the excitability of nerve by blocking of voltage-activated sodium and/or calcium (type N, L, P/Q, and T) channels, by influencing on GABA receptors via direct positive allosteric modulation of GABA_A_ receptor, or indirectly, by increasing levels of GABA via inhibition of GABA transaminase or GABA transporter. Most AEDs have multiple pharmacological activities, whereas in case of retigabine, the primary mechanism of action is the activation of voltage-gated potassium channels in the brain. However, the knowledge that is limited to the molecular targets based on binding affinity or biofunctional studies is insufficient to predict anticonvulsant activity of new molecule (Brodie et al. 2011; Rogawski 2006). Therefore, searching for new drugs which could ameliorate the quality of the treatment of epilepsy seems to be most purposeful and justified. Careful pharmacological screening of potentially suitable molecules in different groups of compounds is an important part of this process. Our studies have been focused on a group of pyrrolidine-2,5-diones as targets for new antiepileptic drugs. Many of these compounds were effective in animal models of seizures and displayed distinctly better safety profile than clinically relevant AEDs ethosuximide, lacosamide, or valproic acid (Kamiński et al. 2015a; Kamiński et al. 2015b; Obniska et al. 2015; Rybka et al. 2014; Rybka et al. 2015). Recently, the synthesis and evaluation of anticonvulsant activity of 22 new derivatives of 3-methylpyrrolidine-2,5-dione and pyrrolidine-2,5-dione have been reported (Rybka et al. 2014). Five of the tested compounds (**12**, **13**, **15**, **23**, and **24**) appeared to be active in the maximal electroshock (MES) test with ED_50_ values range from 16.13 to 46.07 mg/kg and two of them (**12** and **23**) also in the subcutaneous pentylenetetrazole (s.c.PTZ) test with ED_50_ values 134.0 and 128.8 mg/kg, respectively. Moreover, these two compounds did not impair the motor coordination of mice up to a dose of 500 mg/kg in the rotarod test. Thus, the protective indices (PI) in the MES test were highly favorable for compounds **12** (PI > 31) and **23** (PI > 13) than obtained for reference drug: valproic acid (PI = 1.5), phenytoin (PI = 7.74), and lacosamide (PI = 5.0), (Rybka et al. 2014; Kamiński et al. 2015a). In the s.c.PTZ test, the protective indices were also more beneficial for compounds **12** (PI > 3.73) and **23** (PI > 3.88) than for valproic acid (PI = 1.8) and ethosuximide (PI = 2.5).

Taking into consideration the important role of AEDs in the treatment of neuropathic pain, studies on the search for new AEDs should also take into account the evaluation of their usefulness in the treatment of this type of neurological disorder (Mantegazza et al. 2010; Muthuraman et al. 2014). Considering the aforementioned facts, in the present study, additional experiments were performed to improve knowledge on anticonvulsant activity of selected derivatives. Moreover, antinociceptive activity was evaluated in two models of pain in mice: the hot plate and the formalin tests. For preliminary estimation of other behavioral effects, the influence on locomotor activity of mice was also studied. Taking into consideration that drug safety evaluation plays an important role in the early phase of drug development, especially in the preclinical identification of biologically active compounds, the *Vibrio harveyi* assay was performed to determine mutagenic or antimutagenic properties of selected derivatives. To establish the plausible mechanism of anticonvulsant action for the most active compounds, in vitro binding assays for sodium and L-type calcium channels were carried out.

## Materials and methods

### In vivo experiments

#### Animals

Male CD-1 mice weighing 18–24 g were used in the in vivo experiment. The animals were kept at room temperature of 20 ± 2 °C under standard conditions (12:12 h light-dark cycle, standard pellet diet, tap water). All the experiments were performed between 8 a.m. and 3 p.m. For the experiments, the animals were selected in a random way and sacrificed by cervical dislocation immediately after the assay. The experimental protocol was approved by the Local Ethics Commission for Animal Experiments of the Jagiellonian University in Cracow, and all experiences were conducted in accordance with the 1996 NIH Guide for the Care and Use of Laboratory Animals.

#### Chemicals used in pharmacological assays

**Scheme 1:**
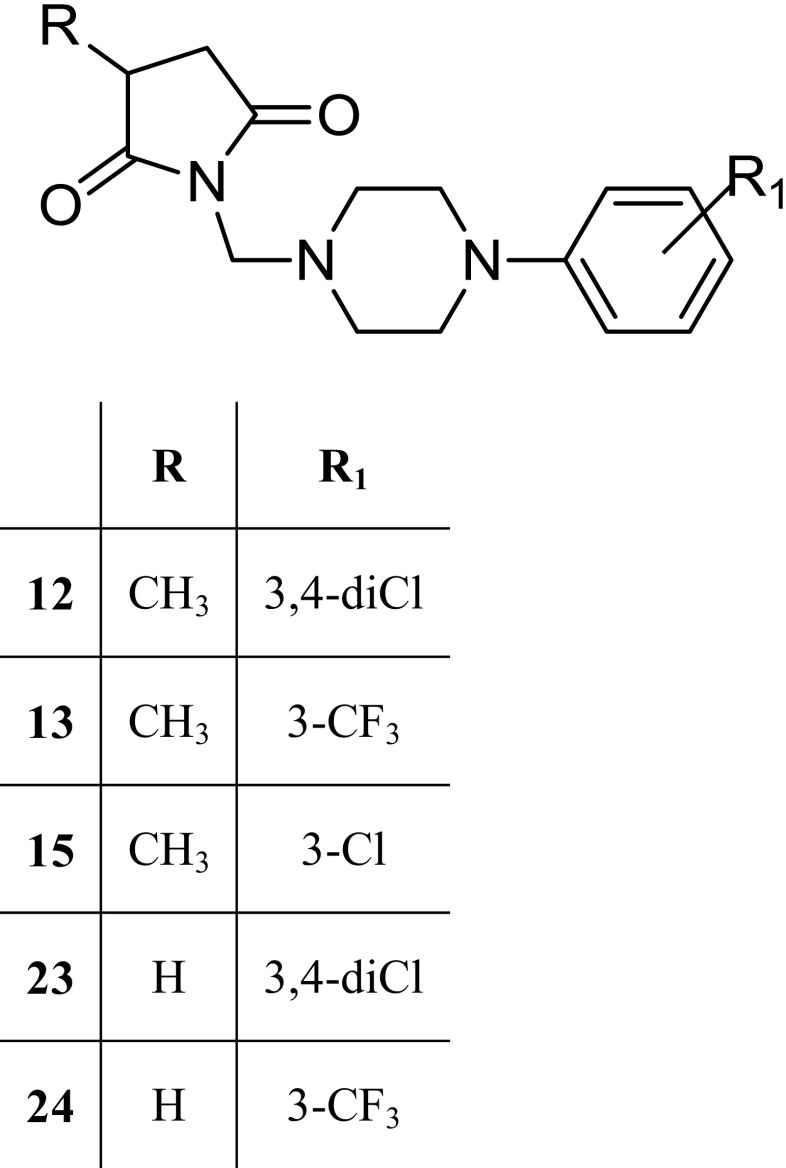
Schematic structure of the studied pyrrolidine-2,5-dione and 3-methylpyrrolidine-2,5-dione

The tested compounds **12** (*N*-[{4-(3,4-dichlorophenyl)-piperazin-1-yl}-methyl]-3-methylpyrrolidine-2,5-dione), **13** (*N*-[{4-(3-trifluoromethylphenyl)-piperazin-1-yl}-methyl]-3-methylpyrrolidine-2,5-dione), **15** (*N*-[{4-(3-methylphenyl)-piperazin-1-yl}-methyl]-3-methylpyrrolidine-2,5-dione), **23** (*N*-[{4-(3,4-dichlorophenyl)-piperazin-1-yl}-methyl]-pyrrolidine-2,5-dione), and **24** (*N*-[{4-(3-trifluoromethylphenyl)-piperazin-1-yl}-methyl]-pyrrolidine-2,5-dione) were synthesized at the Department of Medicinal Chemistry, Jagiellonian University Medical College in Cracow (Scheme 1). The synthesis of the investigated compounds was described earlier (Rybka et al. 2014). The investigated compounds were suspended in a 0.5 % aqueous solution of methylcellulose (Loba Chemie, Germany). Lacosamide (Vimpat, UCB Pharma, Belgium), pilocarpine (Sigma-Aldrich, Germany), scopolamine butylbromide (Sigma-Aldrich, Germany), and formaldehyde (POCh, Poland) were dissolved in distilled water. Agents were administered intraperitoneally (i.p.) at a volume of 0.1 ml/10 g. Control animals were administered an equivalent volume of vehicle (0.5 % aqueous solution of methylcellulose) via the same route as the test compounds. The tested compounds were administered 4 h before the experiments, and lacosamide was administered 30 min before the tests (at the previously established time of peak drug effect). The pretreatment times and doses of tested compounds and lacosamide were established based on previous studies, as well as information about the biological activity of lacosamide taken from the literature (Kamiński et al. 2015a; Rybka et al. 2014; Stöhr et al. 2006; Stöhr et al. 2007).

#### Electroconvulsive threshold test (maximal electroshock seizure threshold test; MEST test)

Electroconvulsions were produced by alternating current (duration of the stimulus 0.2 s; 50 Hz) delivered via auricular electrodes by an electroshock generator (Rodent Shocker, Type 221, Hugo Sachs, Germany) according to the procedure described elsewhere (Löscher et al. 1991; Sałat et al. 2012). Full tonic extension of both hind limbs was taken as the endpoint. Mice not displaying hind limb tonic extension were considered protected from seizure. The convulsive threshold was evaluated as CS_50_, defined as current strength (in mA) required to induce tonic hind limb extension in 50 % of the animals tested, using log-probit method (Litchfield and Wilcoxon 1949). For the evaluation of electroconvulsive threshold, at least three groups of mice (6–8 animals per group) were used.

#### Pilocarpine-induced seizure test

The experimental procedure was originally described by Turski et al. (1987). Seizures were induced in mice by i.p. injection of pilocarpine at a dose of 400 mg/kg. Forty-five minutes before pilocarpine, animals were also pretreated with scopolamine butylbromide (1 mg/kg, i.p.) to reduce peripheral autonomic side effects and masticatory and stereotyped movements (Kowalczyk et al. 2014). Direct observation was performed for 60 min to monitor the latency time to the onset of the limbic seizures, characterized by a subsequent development of behavioral symptoms, such as tremor, head bobbing, and myoclonic movements of the forelimbs progressing to recurrent myoclonic convulsions with rearing, salivation, falling, and status epilepticus (Łuszczki et al. 2008; Turski 2000).

#### The hot plate test

In the hot plate test, the mice were i.p. pretreated either with the test compound or vehicle before placing the animal on the hot plate apparatus (Panlab/Harvard Apparatus, Spain). This apparatus has an electrically heated surface and is supplied with a temperature controller that maintains the temperature at 55–56 °C. The time to the first reaction (licking or jumping) of mice to a nociceptive stimulus was recorded. The cutoff time was established to 60 s to avoid paw tissue damage (Sałat et al. 2014).

#### The formalin test

Antinociceptive activity in the formalin hind paw test was examined according to Laughlin et al. (2002). Twenty microliters of a 5 % formalin solution was injected intraplantarly (i.pl.) into the right hind paw of the mouse. Immediately after formalin injection, the animals were placed individually into glass beakers and were observed for the next 30 min. Time (in seconds) spent on licking or biting the injected hind paw in selected intervals, 0–5 and 15–30 min, was measured in each experimental group and was an indicator of nociceptive behavior. In mice, intraplantarly injection of diluted formalin produces a biphasic nocifensive behavioral response (i.e., licking or biting the injected hind paw). The acute nociceptive phase lasts for the first 5 min, whereas the second inflammatory phase occurs between 15 and 30 min after formalin injection.

#### Spontaneous locomotor activity

Locomotor activity was measured with photoresistor actometers (Ugo Basile, Italy) connected to a counter for the recording of light-beam interruptions. Mice were individually placed in plastic cages for 30 min habituation period, and then the number of light-beam crossings was counted for a 30-min session. The cages were cleaned up with 70 % ethanol after each mouse.

### In vitro experiments

Two *V*. *harveyi* strains were used in the experiments: wild-type BB7 and genetically modified BB7XM (the UV-hypersensitive strain bearing plasmid pAB91273), which were described previously by Czyż et al. (2000). The standard mutagen used as a positive control was 4-nitroquinoline-*N-*oxide (NQNO). The final concentration of NQNO solution used in the tests was 40 ng/ml.

#### Mutagenicity assay

Mutagenicity assay was performed as described previously (Czyż et al. 2000). All experiments were carried out in triplicate and the results were expressed as mutagenic index (M.I. = the number of revertant colonies induced in the tested sample/the number of spontaneous revertants in the negative control) (Gulluce et al. 2010). A compound was considered mutagenic when the M.I. was equal or greater than 2.

#### Antimutagenicity assay

Antimutagenicity assay was performed according to previously described procedures (Czyż et al. 2002; Pękala et al. 2013). All experiments were analyzed in three independent repetitions and the results were expressed as percent inhibition (the ability of the compounds to inhibit the action of NQNO). This was calculated as follows: Inhibition (%) = 100 − [(R1/R2) × 100], where R1 is the number of revertants per plate induced by test compound plus mutagen and R2 is the number of revertants in the plate containing only the mutagen (Słoczyńska et al. 2010). No antimutagenic effect was considered when the inhibition was less than 25 %; a moderate effect for an inhibition value between 25 and 40 % and a strong antimutagenicity were recorded for inhibition higher than 40 % (Gulluce et al. 2010).

#### Sodium and calcium channels binding studies

The radioligand binding studies were performed commercially by Cerep (Celle I’Evescault, France). The binding assays for sodium channel (site 2) was performed using the [^3^H]batrachotoxin and for L-type calcium channel using [^3^H]nitrendipine as radioligands. Compound binding was expressed as a percentage of inhibition of the binding of a radioactively labeled ligand.

#### Statistics

The log-probit method described by Litchfield and Wilcoxon (1949) was used to establish a median current strength (CS_50_) value with the 95 % confidence limits. All the data are presented as the mean ± SEM. Results were statistically evaluated using Student’s *t* test or the one-way analysis of variance (ANOVA) followed by Dunnett’s post hoc comparison test. *P* < 0.05 was considered statistically significant.

## Results

### Influence on the seizure threshold in the MEST test

At a dose of 30 mg/kg, all test compounds caused elevated electroconvulsive threshold (ECT) in comparison to vehicle-treated mice. At this dose, the highest activity revealed compounds **12** and **24**, which increased ECT by 118 % (*p* < 0.01) and 114 % (*p* < 0.001), respectively. The other agents were able to elevate ECT by 24–61 %. At a dose of 10 mg/kg, compounds **12** and **24** increased ECT by 51 % (*p* < 0.001) and 68 % (*p* < 0.001), respectively. Significant activity revealed also compound **23**, since it increased threshold by 61 % (*p* < 0.0001) at a dose of 30 mg/kg and by 27 % (*p* < 0.01) at a dose of 10 mg/kg. Reference drug lacosamide at a dose of 10 mg/kg proved to be highly effective in electrically induced seizures, since it increased threshold for electroconvulsions above 25 mA (Table 1).

**Table 1:** Effect of the tested and reference compounds on the electroconvulsive threshold

Cmpd	Dose (mg/kg)	CS50 (mA) (CI)	SE
Vehicle	–	6.53 (6.11–6.99)	0.22
12	30	14.27 (8.21–24.80)**	2.56
13	30	9.37 (5.47–16.07)*	1.60
15	30	8.11 (7.12–9.24)***	0.43
23	30	10.53 (8.06–13.77)****	0.91
24	30	14.01 (9.05–21.70)***	2.24
Vehicle	–	5.27 (4.35–6.37)	0.33
12	10	7.94 (6.57–9.57)***	0.50
Vehicle	–	6.61 (6.38–6.85)	0.23
23	10	8.42 (7.55–9.39)**	0.39
24	10	11.11 (8.12–15.20)***	1.27
Lacosamide	10	>25	–

*CI* confidence interval, *SE* standard errors

Data analyzed by log-probit method according to Litchfield and Wilcoxon. Result presented as median current strengths (CS_50_) required to evoke tonic hind limb extension in 50 % of mice tested. The compounds were administered i.p. 4 h before the test. Lacosamide was administered i.p. 30 min, before the test

*p < 0.05, **p < 0.01, ***p < 0.001, ****p < 0.0001 vs vehicle; *N* = 18–24

### Anticonvulsant activity in the pilocarpine-induced seizure test

At a dose of 100 mg/kg, two compounds, **12** and **23**, in a statistically significant manner prolonged the latency time to seizures by 37 % (*p* < 0.05) and by 95 % (*p* < 0.001), respectively. Lacosamide at a dose of 40 mg/kg, which was the highest dose without neurotoxic effects in mice, also revealed antiseizure activity, since it prolonged latency time to seizures by 50 % (*p* < 0.05) (Table 2).

**Table 2:** Anticonvulsant activity of the tested and reference compounds in pilocarpine-induced seizures model

Cmpd	Dose	Latency to status epilepticus (s) ± SEM	Anticonvulsant effect (%)
Vehicle	–	342.6 ± 38.15	–
12	100	468.8 ± 39.60*	36.8
13	100	296.7 ± 9.63	–
15	100	406.0 ± 59.77	18.5
23	30	465.8 ± 37.61	36.8
	100	669.4 ± 73.04***	95.4
24	100	346.7 ± 25.92	1.2
Vehicle		338.8 ± 37.85	–
Lacosamide	30	382.1 ± 22.86	12.8
	40^a^	507.7 ± 52.11^*^	49.8

Each value represents the mean ± SEM obtained from 6 to 8 mice. Anticonvulsant activity compared to vehicle-treated mice. Statistical analysis: Student’s *t* test (**12**, **13**, **15**, and **24**) and one-way analysis of variance (ANOVA), followed by Dunnett’s post hoc test (**23** and lacosamide): *F* [2, 19] = 15.72, *p* < 0.001 (**23**); *F* [2, 18] = 4.995, *p* < 0.05 (lacosamide). The compounds were administered i.p. 4 h; lacosamide was administered i.p. 30 min before pilocarpine

*p < 0.05, **p < 0.01, ***p < 0.001 vs vehicle; *N* = 6–8

^a^The highest dose of lacosamide without neurotoxic effects in mice

### Antinociceptive activity in the hot plate test

**Fig. 1:**
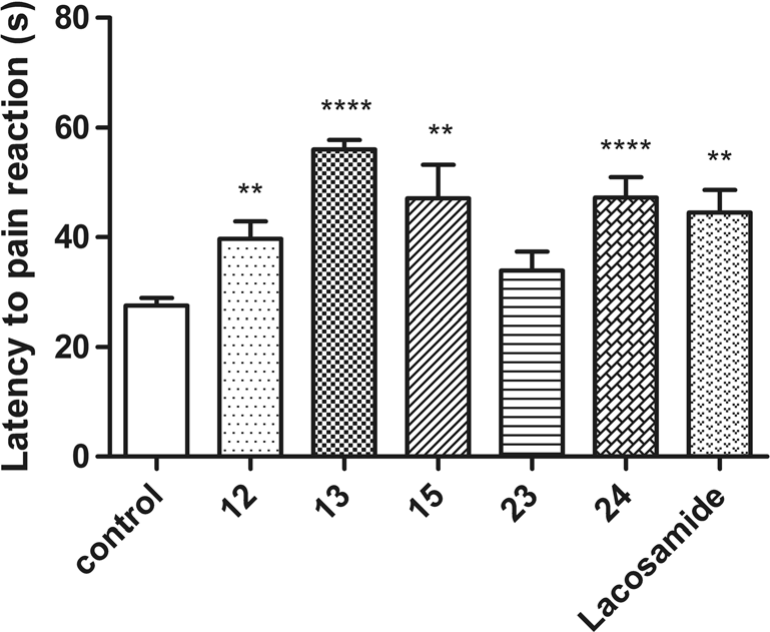
Effects of compounds **12**, **13**, **15**, **23**, **24**, and lacosamide at the dose of 30 mg/kg on response latency in the hot plate test in mice. Data are presented as mean ± SEM and were analyzed by Student *t* test. Significant difference compared to the vehicle-treated group—**p < 0.01, ****p < 0.0001; *N* = 8–10

A significant antinociceptive activity was observed for four of the test compounds: **12**, **13**, **15**, and **24**. Only compound **23** did not show activity in the test. The greatest effect possessed compound **13**, which at a dose of 30 mg/kg significantly prolonged the latency time to pain reaction from 27.51 ± 1.36 s (vehicle-treated group) to 56.03 ± 1.75 s (by 104 %, *p* < 0.0001). Also compounds **15** and **24** displayed a potent effect, as they significantly prolonged the latency time to pain reaction from 27.51 ± 1.36 to 47.12 ± 6.14 s (by 71 %, *p* < 0. 01) and 47.23 ± 3.77 s (by 72 %, *p* < 0.0001), respectively. Compound **12** prolonged the latency time to pain reaction to 39.73 ± 3.20 s (by 44 %, *p* < 0.01). The results are presented in Fig. 1.

### Antinociceptive activity in the formalin hind paw test

**Fig. 2:**
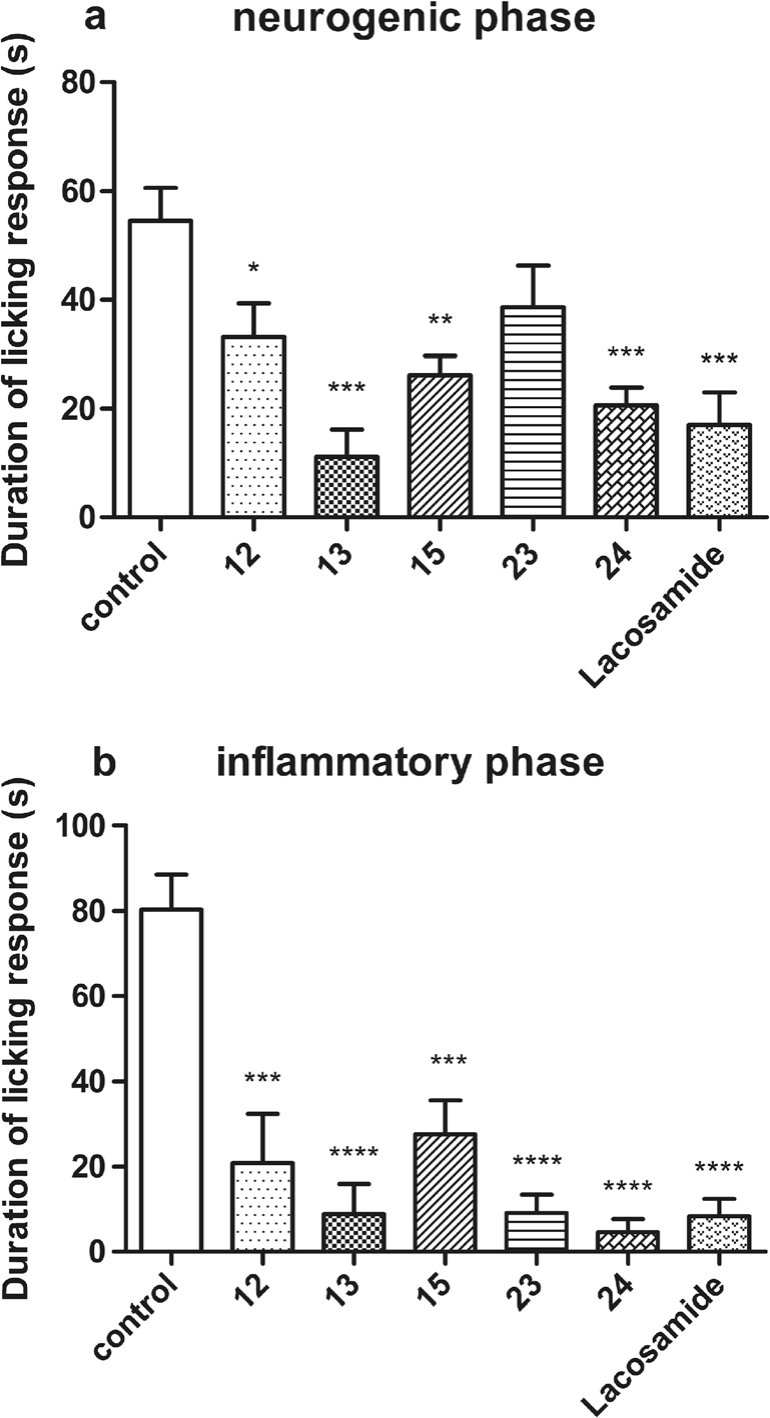
Effects of compounds **12**, **13**, **15**, **23**, **24**, and lacosamide at the dose of 30 mg/kg on the duration of the licking response in **a** the early (neurogenic) phase (0–5 min) or **b** the late (inflammatory) phase (15–30 min) of the formalin test. Data are presented as mean ± SEM and were analyzed by Student *t* test. Significant difference compared to the vehicle-treated group: *p < 0.05, **p < 0.01, ***p < 0.001, ****p < 0.0001; *N* = 8–10

The formalin test was used as a tonic model of nociception. A significant antinociceptive activity was observed for each of the test compounds, given at a dose of 30 mg/kg. In the first (neurogenic) phase of the test, four of the tested compounds significantly reduced the duration of the licking response by 39 % (*p* < 0.05)—**12,** 79 % (*p* < 0.001)—**13,** 52 % (*p* < 0.01)—**15**, and 62 % (*p* < 0.001)—**24**. Only compound **23** did not attenuate the nocifensive response in this phase in a statistically significant manner. In the same phase, lacosamide at a dose of 30 mg/kg displayed antinociceptive effect, as it significantly decreased the duration of the licking response by 69 % (*p* < 0.001) (Fig. 2).

In the second (late) phase of the formalin test, a prominent statistically significant antinociceptive effect was observed for all compounds: **12** (74 %, *p* < 0.001), **13** (89 %, *p* < 0.0001), **15** (66 %, *p* < 0.001), **23** (89 %, *p* < 0.0001), **24** (94 %, *p* < 0.0001), and lacosamide (90 %, *p* < 0.0001) (Fig. 2).

### Influence on locomotor activity

**Fig. 3:**
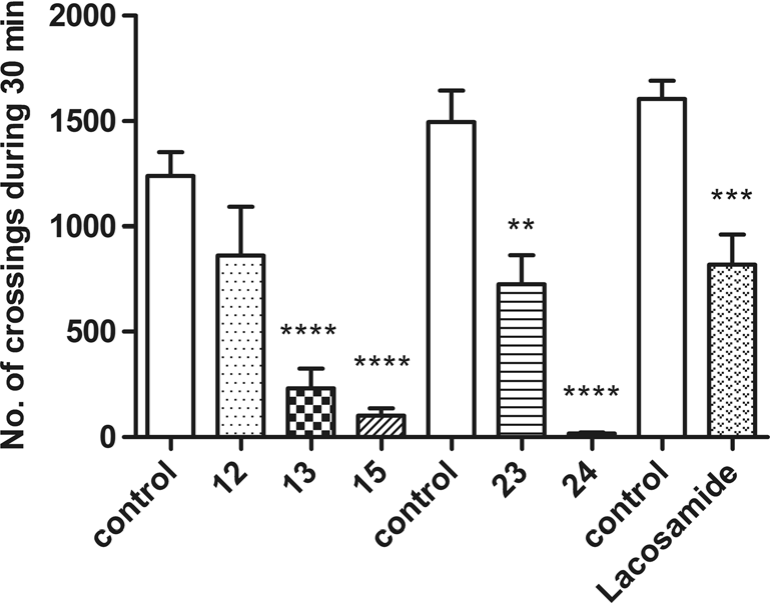
Effects of compounds **12**, **13**, **15**, **23**, **24**, and lacosamide at the dose of 30 mg/kg on locomotor activity in mice. Data are presented as mean ± SEM of beam breaks recorded for 30 min (*n* = 6–10). Statistical analysis of the results was conducted using Student *t* test. Significant difference compared to the vehicle-treated group: **p < 0.01, ***p < 0.001, ****p < 0.0001; *N* = 6–10

Only compound **12** given at a fixed dose of 30 mg/kg did not significantly influence on locomotor activity in mice, while four of the test compounds, **13** (*p* < 0.0001), **15** (*p* < 0.0001), **23** (*p* < 0.01), and **24** (*p* < 0.0001), significantly reduced the number of crossings registered with photoresistor actometers. Also, in the case of lacosamide, a significant decrease of locomotor activity was observed at a dose of 30 mg/kg (*p* < 0.001). The precise data are illustrated in Fig. 3.

### Mutagenic and antimutagenic activity

In the present study, the *V*. *harveyi* assay was used to evaluate mutagenic and antimutagenic properties of four new derivatives of pyrrolidine-2,5-dione (**12**, **13**, **23**, and **24**). The results of the *V*. *harveyi* mutagenicity and antimutagenicity assays for selected derivatives of pyrrolidine-2,5-dione were depicted in Table 3. We found that all tested compounds at a concentration of 40 ng/ml had no mutagenic activity in *V*. *harveyi* BB7 and BB7XM strains. The obtained results show that compound **13** was the most active antimutagenic agent tested. It is worth noting, that compound **13** displayed the highest values of inhibition rates of mutagenicity in all bacterial strains used in the experiment. Two of the compounds investigated, i.e., **12** and **13**, were moderate inhibitors of the mutagenicity induced by the direct-acting mutagen NQNO in *V*. *harveyi* BB7 strain. The inhibition percentages of these substances ranged from 26 to 35, whereas compounds **23** and **24** weakly suppressed the mutagenicity of NQNO in *V*. *harveyi* BB7 strain. The inhibition rates for this compound ranged between 15 and 18 %. Summing up, the most beneficial antimutagenic properties showed compound **13**, which exhibited relatively moderate antimutagenic properties in all tested strains.

**Table 3:** Mutagenicity and antimutagenicity of derivatives of the tested compounds in the *Vibrio harveyi* test

Cmpd	Mutagenicity				Antimutagenicity			
	BB7^a^		BB7XM^a^		BB7^a^		BB7XM^a^	
	Mean ± SD	M.I.^c^	Mean ± SD	M.I.^c^	Mean ± SD	Inhib. (%)^d^	Mean ± SD	Inhib. (%)^d^
DMSO^b^	16 ± 3		12 ± 4		17 ± 2		15 ± 3	
NQNO^b^	32 ± 4	2.0	24 ± 3	2.1	38 ± 5		24 ± 4	
12	25 ± 4	1.5	14 ± 6	1.2	28 ± 4	(26)	23 ± 5	(5)
13	22 ± 3	1.4	20 ± 5	1.7	25 ± 4	(35)	20 ± 4	(16)
23	19 ± 2	1.2	9 ± 3	0.7	32 ± 3	(15)	24 ± 2	(1)
24	25 ± 4	1.6	19 ± 1	1.6	31 ± 5	(18)	29 ± 3	(0)

^a^Number of revertants; ^b^NQNO (nitroquinoline-*N*-oxide, 40 ng/ml) was used as positive control; DMSO was used as negative control; ^c^M.I. (mutagenic index): number of induced revertants/number of spontaneous revertants (positive assay when M.I. ≥ 2); ^d^The values in parenthesis are the inhibition rates (%) of mutagenicity; 25–40 % inhibition: moderate antigenotoxicity, 40 % or more inhibition: strong antigenotoxicity, 25 % or less inhibition: no antigenotoxicity

### In vitro sodium and calcium channel binding studies

Two compounds **12** and **23** which showed prominent anticonvulsant activity and the highest protective indices (PI MES = 31 and 13, respectively) were examined in the binding studies. According to the method applied, compound binding was calculated as a % inhibition of the binding of a radioactively labeled ligand specific for each target. The results are shown in Tables 4 and 5. The tested compounds at a concentration of 100 μM revealed high inhibition of Na^+^ channel (site 2), as it is indicated by inhibition greater than 50 %. At a concentration of 10 μM, these derivatives revealed moderate activity (compound **12—**49.1 % and compound **23**—49.4 %) (Table 4). It should be highlighted that compounds **12** and **23** were stronger Na^+^ channel blockers than phenytoin and carbamazepine, which are known to be the anticonvulsants that act as sodium channel blockers.

**Table 4:** In vitro Na^+^ channel (site 2) binding assays

Cmpd	Concentration (μM)	% inhibition of control specific binding
12	10	49.1
	100	85.8
23	10	49.4
	100	89.9
Phenytoin^a^	10	10.6
	100	21.2
Carbamazepine^b^	10	4.6
	100	17.4

Compounds were each evaluated in preparations from rat cerebral cortex as inhibitors of the specific binding of [^3^H]batrachotoxin to the voltage-sensitive sodium channel. Results showing an inhibition higher than 50 % are considered to represent significant effects of the test compounds; results showing an inhibition between 25 and 50 % are indicative of moderate effect; results showing an inhibition lower than 25 % are not considered significant

^a^Data from the previous study (Rybka et al. 2015)

^b^Data from the previous study (Kamiński et al. 2015b)

**Table 5:** In vitro L-type Ca^+^ (dihydropyridine site) channel binding assays

Cmpd	Concentration (μM)	% inhibition of control specific binding
12	10	20.2
	100	59.4
23	10	12.3
	100	65.9
Phenytoin	10	6.6
	100	57.8
Topiramate^a^	100	7.9

Compounds were each evaluated in preparations from rat cerebral cortex as inhibitors of the specific binding of [^3^H]nitrendipine to the L-type calcium channel. Results showing an inhibition higher than 50 % are considered to represent significant effects of the test compounds; results showing an inhibition between 25 % and 50 % are indicative of moderate effect; results showing an inhibition lower than 25 % are not considered significant

^a^Data from the previous study (Kamiński et al. 2015b)

Moreover, both compounds **12** and **23** were observed as relatively effective binders to L-type calcium channels at a concentration of 100 μM, as they showed inhibition higher than 50 %**—**compound **12** blocked calcium channels in 59.4 % while compound **23** in 65.9 %. Reduction of the concentration to 10 μM resulted in a significant decrease in interaction of compounds with voltage-gated L-type calcium channel—compound **12** blocked this channel in 20.2 % whereas compound **23** in 12.3 % (Table 5). The results of the tested compounds are similar to those obtained for phenytoin. Topiramate, which is the novel AED with a broad therapeutic spectrum, at a concentration of 100 μM was a much weaker binder to the L-type calcium channel than the tested compounds. The findings from the binding studies suggest that the mechanism of anticonvulsant action of compounds is connected with the inhibition of sodium and calcium voltage-gated channels. It is notably visible at a concentration of 100 μM, since compounds **12** and **23** showed binding to both sodium and calcium channels higher than in 50 %.

## Discussion

In this paper, extended studies regarding anticonvulsant and antinociceptive activities of new pyrrolidine-2,5-dione and 3-methylpyrrolidine-2,5-dione are demonstrated. Here, we show that all tested compounds at the dose of 30 mg/kg were able to increase the seizure threshold in the electroconvulsive threshold test. This test, as well as maximal electroshock seizure model (MES test), is regarded to be an experimental model of tonic-clonic seizures and, to a certain extent, partial convulsions with or without secondary generalization in humans (Löscher et al. 1991). Furthermore, the electroconvulsive threshold test can be useful for detecting anticonvulsant agents that act as voltage-gated sodium channel blockers. In the aforementioned experiment, compounds **12**, **23**, and **24** were the most active, as they most considerably elevated the threshold for electroconvulsions in mice, which confirms their excellent anticonvulsant activity in the electrically induced seizures. It is worth noting that the threshold for electroconvulsions in mice seems to be more sensitive than traditional maximal electroshock-induced seizure test as regards the detection of the anticonvulsant properties of antiepileptic drugs (Łuszczki et al. 2007).

The pilocarpine-induced seizure model of epilepsy, originally described by Turski et al. (1987). is a rodent model of status epilepticus. In this test, anticonvulsant drugs including diazepam, clonazepam, valproic acid, and phenobarbital prevent pilocarpine-induced convulsions (Łuszczki et al. 2008). In the previous study, tested compounds proved to be more active in electrically induced (MES test) than in chemically induced seizures (s.c.PTZ test). Therefore, in the pilocarpine-induced seizures, tested compounds were examined at a fixed dose of 100 mg/kg. The significant activity displayed only two compounds: **12** and **23**. These compounds were also active in electrically induced seizures and pentylenetetrazole-induced seizures (previous study); therefore, they seem to be molecules which display broad spectra of anticonvulsant effects. Lacosamide, a novel anticonvulsant drug tested at a dose of 40 mg/kg (the highest dose that did not cause neurotoxic effects in mice), also significantly prolonged latency time to status epilepticus in aforementioned animal model of seizures. Currently this drug, as well as other second-line drugs, such as levetiracetam, topiramate, or pregabaline have been increasingly used for the treatment of status epilepticus in humans (Kellinghaus et al. 2014; Rossetti and Lowenstein 2011).

It is well known that antiepileptic drugs (AEDs) are used not only to treat epilepsy but also to treat diverse non-epileptic conditions, including pain (neuropathic pain, migraine prophylaxis), neuromuscular disorders, and psychiatric disorders (anxiety, bipolar affective disorders) (Mantegazza et al. 2010; Rogawski and Löscher 2004). Studies by various groups using different models of pain suggest that antiepileptic drugs can inhibit sensitized signaling associated with allodynia and hyperalgesia (Beyreuther et al. 2006; Laughlin et al. 2002; Sałat et al. 2014).

Therefore, the objective of this study was also to characterize the antinociceptive activity of the tested compounds. This activity was investigated in mice in the hot plate test and the formalin test. The hot plate test is widely used for evaluation of the centrally acting analgesics. The mechanism of analgesic effect observed here is found to be a consequence of supraspinal attenuation of ascending nociceptive input (Listos et al. 2014; Manzanares et al. 2006). In the present study, four of the tested compounds, **12**, **13**, **15**, and **24**, as well as lacosamide at a dose of 30 mg/kg, significantly prolonged the latency time for reaction responses. The highest activity showed compound **13**, which prolonged the latency time to pain reaction by about 104 %.

Many studies have demonstrated that compounds which revealed anticonvulsant activity in the MES test exhibit an antinociceptive action in the formalin test in mice. The injection of formalin to a mouse paw which results in neuronal damage produced two phases of pain response: the early–acute chemical pain and the late–tonic nociception involving central sensitization (Laughlin et al. 2002). It has been reported that the late phase is either the effect of sensitization of the dorsal horn neurons of the spinal cord or inflammation-induced hyperactivity of afferent nociceptors or combination of both (Ximenes et al. 2013). For compounds **12**, **13**, **15**, and **24**, as well as lacosamide at the dose of 30 mg/kg, the significant analgesic effect was observed in the early (neurogenic) phase of the test. Whereas compound **23**, likewise in the hot plate test, did not reveal significant analgesic activity in the first phase. In this phase of formalin test, likewise in the hot plate test, the most active was compound **13**. These results indicate that this compound exerts a prominent central antinociceptive effect. In the second phase of the formalin test, all of tested compounds, as well as lacosamide, revealed a significant antinociceptive activity. Lacosamide displayed an analgesic effect in this test also in research conducted by other groups (Beyreuther et al. 2007; Stöhr et al. 2006).

The obtained results of anticonvulsant and antinociceptive activity are very promising. However, compounds **13**, **15**, **23**, and **24** significantly altered the locomotor activity of mice; thus, drug-induced sedation can impede the use of these compounds as potential drugs. Hence, compound **12**, with remarkable anticonvulsant and antinociceptive properties, which did not produce any significant effects on the locomotor activity, as well as the motor coordination in rotarod test (even at high doses TD_50_ > 500 mg/kg), seems to be the most promising compound, thus prompting further studies to confirm its safety and efficacy.

In the *V*. *harveyi* assay, the studied agents did not display mutagenic activity. It is noteworthy that in the case of derivative **13**, its antimutagenic activity against 4-NQO implies that this compound may directly protect against DNA damage from mutagens. Additionally, it appears that the *V*. *harveyi* assay can be applied for primary mutagenicity and antimutagenicity assessment of chemical substances, thus representing a useful alternative tool for compound safety evaluation.

In spite of intensive research on the physiological and biochemical bases of epilepsy, its pathogenesis is not completely understood; however, it is known that the cause of seizures can be malfunctioning of both sodium and calcium channels. The voltage-gated sodium channels have been the target of many antiepileptic drugs, including phenytoin, lamotrigine, and carbamazepine. Moreover, lacosamide has been found also as a voltage-gated sodium channel inhibitor, but it differs from other AEDs in the fact that it selectively enhances the slow inactivation without affecting the fast inactivation of these channels (Perucca et al. 2007). Like other voltage-gated ion channels, voltage-gated Ca^2+^ (L-, P/Q-, N-type) channels play an essential role in proper functioning of neurons. The well-known AEDs which influence L-type calcium channel activity are phenytoin, topiramate, and felbamate (Liu et al. 2003; Rogawski and Löscher 2004). Taking into account the above remarks, the influence on the neuronal voltage-sensitive sodium and L-type calcium channels was determinated for two compounds, **12** and **23**, which showed prominent anticonvulsant activity with high protective indices. At a concentration of 100 μM, these selected active molecules inhibited sodium and calcium voltage-gated channels in highly more than 50 %, which is a better result than for the reference drugs**—**phenytoin, carbamazepine, and topiramate. At a concentration of 10 μM, they still revealed a moderate effect on sodium and calcium channels. It should be highlighted that derivatives **12** and **23** were stronger sodium (site 2) and L-type calcium channel blockers than reference drugs.

## Conclusions

In conclusion, we demonstrated by several in vivo experimental models prominent anticonvulsant and analgesic effects of new derivatives of pyrrolidine-2,5-dione and 3-methylpyrrolidine-2,5-dione. The most promising effect was observed for compounds **12** and **23** which displayed broad spectra of anticonvulsant activity. At active doses in experimental tests, compound **12** did not show sedative properties. Moreover, the tested molecules did not display mutagenic activity in the *V*. *harveyi* assay. The in vitro binding study indicated that the plausible mechanism of action of compounds **12** and **23** was the influence on the neuronal voltage-sensitive sodium (site 2) and L-type calcium channels. The results obtained in the current studies proved that in the group of pyrrolidine-2,5-diones, new anticonvulsants with collateral analgesic properties can be found.
